# Patients Report A Positive Experience on Social Media After Bankart Repair

**DOI:** 10.1016/j.asmr.2022.03.011

**Published:** 2022-06-04

**Authors:** Sanchita Gupta, Wendell W. Cole, Cadence Miskimin, Michaela Stamm, Mary K. Mulcahey

**Affiliations:** a Tulane University School of Medicine, New Orleans, Louisiana, U.S.A; b Tulane University School of Medicine, Department of Orthopaedic Surgery, New Orleans, Louisiana, U.S.A

## Abstract

**Purpose:**

The purpose of this cross-sectional study was to analyze publicly available posts on Instagram and Twitter to gain an understanding of patients’ perspectives regarding Bankart injuries and repair.

**Methods:**

Public posts on Instagram and Twitter were queried from June 1, 2019, to June 1, 2020, with the following hashtags: #Bankart #Bankartrepair #Bankartlesion #labrumrepair #labralrepair #shoulderdislocation. Posts that did not contain those hashtags were excluded. In addition, posts that included that hashtag but displayed content unrelated to Bankart repair were excluded. A binary categorical system was used for media format (picture or video), perspective (patient, family or friend, physician, hospital or physical therapy group, professional organization, news media, or industry), timing (preoperative, postoperative, nonoperative), tone (positive, negative, or neutral), content (surgical site, hospital or surgeon, imaging, rehabilitation, activities of daily living [ADLs], return to work, surgical instruments, or education), post popularity (number of likes), and geographic location.

**Results:**

1,154 Instagram posts were identified. 722/1,154 posts (62.6%) were made by patients. 600 (52.0%) of the post tones were positive, 407 (35.3%) were neutral, and 667 (57.8%) were postoperative. The most common content included in Instagram posts were ADLs (577; 50.0%), education (233; 20.2%), and rehabilitation (226; 19.6%). Overall, posts had an average of 117 likes and had geotags from 49 different countries. 155 tweets were identified, 92 of which (59.4%) were made by physicians, 113 (72.9%) were neutral, 127 (81.9%) were nonoperative, and the most common type of content posted was education (130; 83.9%). Overall, posts on Twitter had an average of 3.2 likes and had geotags from 4 different countries.

**Conclusions:**

Instagram posts were made mostly by patients postoperatively and focused on ADLs. The tone of the Instagram posts indicates that a majority of patients have a positive experience with Bankart repair. The majority of tweets were made by physicians and provided educational information with a neutral tone.

**Clinical Relevance:**

Exploring patient's experiences with Bankart repair on social media provides insight into their overall experience with the surgery. The majority of patients reported a positive experience.

## Introduction

Bankart lesions are considered the essential lesion of shoulder instability and frequently occur following anterior shoulder dislocation in young patients. In the past 30 years, because of technological advances, arthroscopic Bankart repair has become the primary technique used in treating recurrent anterior shoulder instability.^1^ In athletes, arthroscopic shoulder stabilization has been performed for recurrent shoulder instability with excellent results, low revision rates, and high levels of patient satisfaction.^2^, ^3^, ^4^ Despite these results, the recurrence rate of shoulder instability in patients who have had previous arthroscopic shoulder stabilization has been found to range from 9.7 to 22%.^3^^,^^5^ Different tools such as postoperative surveys can be used to assess patient satisfaction. However, in the past several years, social media have offered an outlet for patients to document their unbiased surgical experience. Therefore, social media can be used as a tool to analyze patient perception and satisfaction following Bankart repair.

Prior studies have examined the perspective of patients, surgeons, and hospitals through social media platforms, such as Instagram and Twitter. Haeberle et al. used Instagram to examine patient perception of hip arthroscopy and found that of the 1,850 posts analyzed, 91.2% were made by patients, and 52.9% were positive.^6^ Ramkumar et al. used Twitter and Instagram to examine the nature of social media content related to shoulder and elbow surgery, including shoulder arthroplasty and rotator cuff repair.^7^ The purpose of this cross-sectional study was to analyze publicly available posts on Instagram and Twitter to gain an understanding of patients’ perspectives regarding Bankart injuries and repair. We hypothesized that the majority of social media posts would be made postoperatively by patients in a positive tone.

## Methods

Public posts on Instagram and Twitter made from June 1, 2019, to June 1, 2020, were identified using the following hashtags: #Bankart #Bankartrepair #Bankartlesion #labrumrepair #labralrepair, and #shoulderdislocation. Instagram and Twitter were specifically chosen because of the user-friendly interface that allowed for the easy filtering of posts by hashtag and chronological date, concurrently. All posts relating to human subjects were included. All veterinary and non-human content was excluded, as well as posts discussing other procedures. Posts were also excluded if they were not in English, to avoid misinterpretation.

### Analysis

Data were collected and analyzed in Microsoft Excel. A binary scoring system was used for media format, perspective, timing, perioperative period, tone, content, post popularity, perspective (patient, physician, hospital, non-medical employee), and geographic location.

## Results

### Instagram

Of the total 1,154 Instagram posts, the majority of posts do not use the term Bankart, but rather, 503 (43.5%) used the hashtag #shoulderdislocation and 393 (34.1%) used #labrumrepair

Three hundred twenty-one of “1,154” Instagram posts (81.7%) used #labrumrepair, 49 (77.7%) used #labralrepair, and 388 (77.1%) used #shoulderdislocation (Table 2). The majority of Instagram posts did not directly report Bankart injury, but rather, it was referenced using the hashtags such as #bankartrepair, #labrumrepair, and #labralrepair.

**Table 1:** Instagram and Twitter Posts by Hashtag

Hashtag	Number of Instagram Posts (% of Total)	Number of Twitter Posts (% of Total)
#shoulderdislocation	503 (43.5)	92 (59.4)
#labrumrepair	393 (34.1)	6 (3.9)
#Bankartrepair	100 (8.7)	20 (12.9)
#Bankart	81 (7.0)	30 (19.4)
#labralrepair	63 (5.5)	5 (3.2)
#Bankartlesion	14 (1.2)	2 (1.3)
Total Posts	1,154	155

**Table 2:** Instagram and Twitter Injury Categorization by Hashtag

Injuries Under #labrumrepair (393)	Instagram *N* (% of Total Number of #labrumrepair)	Number of posts from Instagram from patients	Twitter *N* (% of Total Number of #labrumrepair)	Number of Posts From Twitter From Patients
Not reported	321 (81.7)		5 (83.3)	
SLAP tear + Posterior labral tear	38 (9.7)	38		
SLAP tear	23 (5.8)	18	1 (16.7)	0
SLAP tear + Bankart	10 (2.5)	10		
Posterior labral tear	1 (0.3)	1		
Injuries under #labralrepair (63)	*N* (% of Total Number of #labralrepair)		*N* (% of Total Number of #labralrepair)	
Not reported	49 (77.7%)		5 (100)	
SLAP Tear	5 (7.9%)	3		
Anterior labral tear	2 (3.2%)	0		
Bankart	2 (3.2%)	0		
Posterior labral tear	2 (3.2%)	0		
Anterior and posterior labral tear	1 (1.6%)	0		
360° Labral tear	1 (1.6%)	0		
SLAP tear + Bankart	1 (1.6%)	1		
Injuries under #shoulderdislocation (503)	*N* (% of Total Number of #shoulderdislocation)		*N* (% of Total Number of #shoulderdislocation)	
Not reported	388 (77.1)		76 (82.6)	
Anterior dislocation	79 (15.7)	8	4 (4.4)	0
Multidirectional instability	15 (3.0)	5		
Both anterior and posterior	11 (2.2)	0	8 (8.7)	0
Posterior dislocation	10 (2.0)	0	4 (4.4)	0

A total of 1,154 posts were included in the analysis. The media format of 647 (56.1%) of the posts were pictures and 507 (43.9%) were videos. The majority of Instagram posts were from the patient perspective (722; 62.6%), while 238 (20.6 %) were from hospitals or physical therapists, and 134 (11.6%) were from physicians. In regard to timing of posts, 37 (3.2%) were preoperative, 86 (7.5%) were perioperative, 667 (57.8%) were postoperative, and 364 (31.5%) were nonoperative. The content types found were: ADL: 577 (50.0%), education: 233 (20.2%), rehabilitation: 226 (19.6%), hospital or surgeon: 46 (3.9%), wound site: 29 (2.5%), other: 22 (1.9%), imaging: 9 (0.8%), instrument or device 9 (0.8%), and return to work 3 (0.3%). A complete summary of the Instagram content can be found in Table 3.

**Table 3:** Summary of Instagram and Twitter Content

Overall Results		Instagram *N* (%) out of Total 1,154 posts	*N* (% of Total) out of Total 155 Tweets
Media format	Picture	647 (56.1)	113 (72.9)
	Video	507 (43.9)	18 (11.6)
	Tweet		24 (15.5)
Perspective (post author)	Patient	722 (62.6)	15 (9.7)
	Hospital or physical therapy group	238 (20.6)	11 (7.1)
	Physician	134 (11.6)	92 (59.4)
	Professional organization	37 (3.2)	36 (23.2)
	Family or friend	13 (1.1)	
	News media	5 (0.4)	
	Industry	5 (0.4)	
	PhD		1 (0.7)
Timing	Preoperative	37 (3.2)	3 (1.9)
	Perioperative (from the time of surgery to 1 month postoperatively)	86 (7.5)	7 (4.5)
	Postoperative	667 (57.8)	18 (11.6)
	Not Reported	364 (31.5)	127 (81.9)
Tone	Positive	600 (52.0)	32 (20.6)
	Neutral	407 (35.3)	113 (72.9)
	Negative	147 (12.7)	10 (6.4)
Content type	Activities of daily living (ADL)	577 (50.0)	11 (7.1)
	Education	233 (20.2)	130 (83.9)
	Rehabilitation	226 (19.6)	5 (3.2)
	Hospital or surgeon	46 (3.9)	6 (3.9)
	Wound site	29 (2.5)	
	Other	22 (1.9)	
	Imaging	9 (0.8)	
	Instrument or device	9 (0.8)	2 (1.3)
	Return to work	3 (0.3)	
	Closed reduction		1 (0.7)
Post popularity	Average likes	118.0 (Range 0–7040)	3.2 (Range 0–59 likes)
Geographic location	Not available	600 (52.0)	77 (49.7)
	North America	398 (34.5)	53 (34.2)
	Asia	74 (6.4)	8 (5.2)
	Europe	48 (4.2)	17 (10.9)
	Australia	28 (2.4)	
	Africa	4 (0.4)	
	South America	2 (0.2)	

Ninety-two of 155 (59.4%) Twitter posts (tweets) were identified using #shoulderdislocation, 30 (19.4%) using #Bankart, 20 (12.9%) using #Bankartrepair, 6 (3.9%) using #labrumrepair, 5 (3.2%) using #labralrepair, and 2 (1.3%) using #Bankartlesion (Table 1).

Under each Twitter hashtag, the majority of tweets did not specify the type of injury. Of the 6 tweets that used #labrumrepair, 5 (83.3%) did not report the type of injury; for #labralrepair, none of the 5 posts (100%) reported the type of injury; for #shoulderdislocation, 76 of 92 tweets (82.6%) did not report the type of injury (Table 2).

The majority of the tweets were in picture format (113 of 155; 72.9%), followed by text (24; 15.5%), and video (18; 11.6%). The most common perspectives of the tweets were from a physician (92; 59.4%) and professional organizations (36; 23.2%). The majority of tweets (127 of 155; 81.9%) did not specify whether they were pre-, peri-, or postoperative. One hundred and thirteen of 155 tweets (72.9%) were neutral in tone, while 32 (20.6%) had a positive tone, and 10 (6.4%) had a negative tone. Education was the most common content (130; 83.9%), followed by activities of daily living (ADLs) (11; 7.1%). The average number of likes for all tweets was 3.2 (range 0–59 likes). Tweets were from 4 different countries, with the majority being from the United States (53 of 155; 34.2%). Table 3 summarizes the analysis of the Twitter content.

## Discussion

This study demonstrated that patients with Bankart injuries focus their social media posts primarily on daily life activities and rehabilitation. Photos were the predominant form of media shared across both Instagram and Twitter. Instagram was used primarily to provide insight into patients’ daily lives and activities, while Twitter was mostly to share educational content. The majority of posts on Instagram were from patients, while on Twitter they were from physicians and tended to focus on education.

Numerous previous studies have examined patient satisfaction of Bankart Repair outside of social media. In 2022, Zink et al. followed a cohort of 49 patients for a 10-year postoperative period and showed that 95.5% of patients were very satisfied or satisfied at the end of the follow-up.^8^ In 2021, Hurley et al. followed 144 patients for a 5-year follow-up and showed 82.6% of patients were satisfied/very satisfied with Bankart Repair.^9^ However, these studies are limited by smaller cohort sizes. Comparatively, our study demonstrated that 52% of Instagram posts had a positive tone. Specifically, 490 patients reported a positive response out of the 601 posts with a positive tone. Among Twitter posts, 8 patients reported a positive response out of the 32 posts with a positive tone.

In addition, there have been multiple studies on the use of social media to evaluate patient perception following orthopaedic injuries in sports medicine, shoulder and elbow surgery, total joint arthroplasty, and spine.^7^^,^^10^, ^11^, ^12^ Our results are similar to a study performed by Haeberle et al., who reviewed 1,850 Instagram posts and 163 tweets related to patient perception of hip arthroscopy and found that approximately 53% had a positive tone.^6^ However, this rate is markedly lower than posts on Instagram and Twitter related to shoulder and elbow surgery (97% positive), anterior cruciate ligament (ACL) reconstruction (88% positive), and total hip and knee joint arthroplasty Instagram posts (93% positive).^1^^,^^5^^,^^13^^,^^14^ In the current study, there was a higher percentage of neutral content compared to previous studies, which may have lowered the relative percentage of positive posts.

Our study also demonstrated that the most common type of Instagram and Twitter content posted was related to ADLs, rehabilitation, and educational topics. These results are similar to a study performed by Rizalla et al., who noted that a majority of Instagram posts related to spinal fusion focused on returning to daily activities.^7^ In 2017, Ramkumar et al. analyzed 3,145 posts related to ACL reconstruction, which were made by patients, surgeons, and hospitals.^9^ The most common types of content were personal recovery stories (92% of overall posts), with an emphasis on wound appearance, the rehabilitation process, and return to play.^11^ These results differ from our study, as only 2.5% of patients posted about their wound appearance. This may be related to the fact that the incisions for arthroscopic Bankart repair are much smaller than those made for ACL reconstruction and can be covered by a shirt, whereas knee incisions are more easily visible when wearing shorts/skirts. In a separate study, Ramkumar et al., used Instagram to examine patient perception of total joint arthroplasty by evaluating 1,287 individual public posts.^10^ The authors found that 91% of posts were shared during the postoperative period, 93% had a positive tone, and the focus of 34% of the posts were on ADLs and 34% were on rehabilitation.^12^

Compared to Instagram posts, tweets were mostly from the perspective of physicians, focused largely on educational content, and were neutral in tone. This is similar to the Haeberle et al. study on social media analysis of hip arthroscopy, which demonstrated that the majority of tweets were made by physicians (50.9%), included educational content (64.4%) and were neutral in tone (44.8%).^6^ When comparing the total post percentages from Twitter vs. Instagram, we found that the physician perspective accounted for 59.4% vs 11.6%, educational content was 83.9% vs 20.2%, and neutral tone was 72.9% vs 35.3%. This suggests that Twitter may be used more for educational purposes by physicians. On the other hand, Instagram may be more patient-centered and allow for more patient-patient interaction, which is also reflected by the substantial difference in average number of likes on Instagram (118) versus Twitter (3.2). In addition, Instagram and Twitter users have the option to report their geographic location through their geotag. The geotag optional feature was used more commonly on Instagram than Twitter, with Instagram representing 39 different countries, compared to Twitter, which represented only 4 different countries.

### Limitations

There are several limitations to this study. First, only #shoulderdislocation, #labrumrepair, #Bankartrepair, #Bankart, #labralrepair, and #Bankartlesion were used. While over 95% of glenohumeral dislocations are anterior, which almost always results in a Bankart lesion, especially in young patients, there is a possibility that the posts with an unspecified type of dislocation may have been posterior shoulder dislocation.^15^ In addition, when Instagram was queried with the hashtags #labralrepair and #labrumrepair, some posts did not specify whether a Bankart lesion was present or not; therefore, it is possible that the patient’s injury may not have been a Bankart lesion. Second, only public posts found on Instagram and Twitter were evaluated. There may have been a number of private posts that contained a relevant hashtag, but these posts were inaccessible due to privacy settings. Third, the primary search of the posts was done using only hashtags. This excluded posts that may have used the same key words found in our hashtag without the use of the hashtag itself. In addition, in the future, posts can be queried using hashtags as single words such as #shoulder #dislocation #bankart #repair.

### Conclusion

Instagram posts were made mostly by patients postoperatively and focused on ADLs. The tone of the Instagram posts indicates that a majority of patients have a positive experience with Bankart repair. The majority of tweets were made by physicians and provided educational information with a neutral tone.

## Supplementary Data

ICMJE author disclosure forms

## References

[1] Fountzoulas K., Hassan S., Khoriati A.A., Chiang C.H., Little N., Patel V. (2020). Arthroscopic stabilisation for shoulder instability. J Clin Orthop Trauma.

[2] Cordasco F.A., Lin B., Heller M., Asaro L.A., Ling D., Calcei J.G. (2020). Arthroscopic shoulder stabilization in the young athlete: Return to sport and revision stabilization rates. J Shoulder Elbow Surg.

[3] Vermeulen A.E., Landman E.B.M., Veen E.J.D., Nienhuis S., Koorevaar C.T. (2019). Long-term clinical outcome of arthroscopic Bankart repair with suture anchors. J Shoulder Elbow Surg.

[4] Moore T.K., Hurley E.T., Rowe D.N. (2020). Outcomes following arthroscopic Bankart repair in female patients. J Shoulder Elbow Surg.

[5] Szyluk K., Jasinski A., Widuchowski W., Mielnik M., Koczy B. (2015). Results of arthroscopic Bankart lesion repair in patients with post-traumatic anterior instability of the shoulder and a non-engaging Hill-Sachs lesion with a suture anchor after a minimum of 6-year follow-up. Med Sci Monit.

[6] Haeberle H.S., Bartschat N.I., Navarro S.M. (2019). Hip arthroscopy: A social media analysis of patient perception. Orthop J Sports Med.

[7] Ramkumar P.N., Navarro S.M., Cornaghie M.M. (2018). Social media in shoulder & elbow surgery: An analysis of Twitter and Instagram. Int J Sports Med.

[8] Zink S, Pfeiffenberger T, Muller A, Krisam R, Unglaub F, Potzl W. The arthroscopic Bankart operation: A 10-year follow-up study. *Arch Orthop Trauma Surg* In press. doi:10.1007/s00402-021-04282-4. 10.1007/s00402-021-04282-4 34999993

[9] Hurley ET, Davey MS, Mojica ES, et al. Evaluation of factors associated with successful 5-year outcomes following arthroscopic Bankart repair in athletes. *Knee Surg Sports Traumatol Arthrosc* In press. doi:10.1007/s00167-021-06803-5. 10.1007/s00167-021-06803-5 34811577

[10] Rizkalla JM, Holderread B, Hotchkiss W, et al. Instagram and spine fusion: An analysis of social media and its relationship to patient perception of surgery. *Global Spine J* In press. doi:10.1177/21925682211001814. 10.1177/21925682211001814 PMC10240586 33787373

[11] Ramkumar P.N., La T., Fisch E. (2017). Integrating social media and anterior cruciate ligament surgery: An analysis of patient, surgeon, and hospital use. Arthroscopy.

[12] Ramkumar P.N., Navarro S.M., Haeberle H.S., Chughtai M., Flynn M.E., Mont M.A. (2017). Social media and total joint arthroplasty: An analysis of patient utilization on Instagram. J Arthroplasty.

[13] Altintas B., Godin J.A., Millett P.J., Millett P.J., Pogorzelski J. (2019). Advanced techniques in shoulder arthroscopy.

[14] Lee D.H., Neviaser R.J. (2010).

[15] Cutts S., Prempeh M., Drew S. (2009). Anterior shoulder dislocation. Ann R Coll Surg Engl.

